# Increased incidence of metachronous gastric neoplasm after endoscopic resection in patients with synchronous gastric neoplasm

**DOI:** 10.1186/s12876-020-01358-0

**Published:** 2020-06-30

**Authors:** Ga-Yeong Shin, Hye Jin Cho, Jae Myung Park, Chul-Hyun Lim, Yu Kyung Cho, Myung-Gyu Choi

**Affiliations:** 1 Division of Gastroenterology & Hepatology, Department of Internal Medicine, Seoul St. Mary’s Hospital, College of Medicine, The Catholic University of Korea, 222 Banpo-daero, Seocho-GU, Seoul, 137-701 South Korea; 2 Catholic Photomedicine Research Institute, The Catholic University of Korea, Seoul, South Korea

**Keywords:** Stomach neoplasms, Endoscopy, Recurrence, Endoscopic mucosal resection

## Abstract

**Background:**

Recurrence risk is a major concern after endoscopic resection (ER) of gastric neoplasms. This study was to compare metachronous risk in patients with and without synchronous neoplasms after complete ER.

**Methods:**

After ER for gastric neoplasms, patients were divided into those with and without synchronous neoplasm. The metachronous risk of gastric neoplasms was compared between the two groups.

**Results:**

After ER of 678 cancers and 891 adenomas, synchronous neoplasm was found in 11.8% of cancers and 11.4% of adenomas. In the multiple (*n* = 182) and the single group (*n* = 1387), metachronous neoplasms occurred in 18.1 and 8.6%, respectively (HR 2.40; 95% CI, 1.62–3.34). When the pathology of the recurred lesion was limited to cancer, metachronous risk was also significantly higher in the multiple than in the single group (HR, 2.2; 95% CI, 1.17–3.85). In the recurred pathology of the multiple group, cancer development was frequently observed in patients with cancer compared to those with only adenomas in the synchronous lesion (67.0% vs. 13.0%, respectively; *P* = 0.023).

**Conclusions:**

This study demonstrated that metachronous risk was significantly higher in patients with synchronous gastric neoplasms after ER. Therefore, meticulous examination is important in patients with synchronous neoplasm.

## Background

Gastric cancer is the fifth most common cancer worldwide, comprising 6.8% of total cancer incidence, and the third leading cause of cancer death, making up 8.8% of total cancer deaths [1]. Therefore, early detection of gastric cancer is important for a better prognosis [2]. In Korea, a screening program has allowed detection of gastric cancer at an early stage, resulting in a reduction in mortality [3].

Endoscopic resection (ER) including endoscopic mucosal resection (EMR) and endoscopic submucosal dissection (ESD) is now widely performed and accepted as an effective treatment option for early gastric cancer (EGC) that poses a low risk of lymph node metastasis [4]. Guidelines also recommend removal of adenomas as well as EGCs for diagnosis and treatment [5, 6], because adenomas are precancerous lesions [7] but initially noninvasive adenomas can advance to invasive adenocarcinoma even after ER in 4 to 30% of cases [8, 9].

Although ER has many advantages including quality of life, cost-effectiveness, and hospital stay compared to surgery, the development of metachronous cancer in the remnant gastric mucosa is one of its major problems [10, 11]. The incidence of metachronous cancer after ER ranges between 2.7 and 14% over 2.2 to 7 years of follow-up [12–14]. Many studies have reported risk factors for metachronous cancer after ER, which included the patient’s age, multiple EGCs, persistent *Helicobacter pylori*infection [13–15]. However, these studies were conducted after removal of EGCs only, not including adenomas.

Considering our previous study report that the incidence of post-ER metachronous gastric cancer was not significantly different after removal of adenomas and EGCs [16], the metachronous risk of gastric adenoma after ER should be investigated as well. Furthermore, the incidence of metachronous neoplasm is not well documented in patients with synchronous gastric neoplasms after removal of lesions, although synchronous gastric cancer has been reported at rates of 5–11% in the resected stomach after ER [17].

In this study, we investigated the incidence of metachronous gastric neoplasm between patients with and without synchronous gastric neoplasms. We also compared the outcomes of metachronous neoplasm according to the histological types of synchronous neoplasms.

## Methods

### Patients

This study was retrospectively performed in patients who underwent endoscopic resection (ER) of gastric neoplasms between January 1999 and December 2015 at Seoul St. Mary’s Hospital in Seoul, Korea. We excluded patients with a history of gastric cancer surgery, additional gastrectomy after non-curative or incomplete ER, no diagnosis of adenoma or EGC after ER, recurrence at previous ER site, and follow-up of less than 1 year. We retrieved medical records which included patients’ demographics, pathology results, ER modalities, infection status of *H. pylori*, gastric atrophy, and the development of metachronous neoplasm. In our study, pathological diagnoses were based on Vienna classification [18, 19]: adenoma group contained LGD as category 3, HGD as category 4.1, and non-invasive carcinoma as category 4.2, suspicion of invasive carcinoma as category 4.3, and invasive neoplasia as category 5 belong to the carcinoma group. We defined neoplasms as synchronous when the lesions detected by endoscopic examination within 12 months from the time of ER. Patients were divided into two groups by the absence (the single group) or presence (the multiple group) of synchronous lesions. Patients in the multiple group were further divided into two subgroups according to having or not having cancer on the resected specimen histology. This study was approved by the Institutional Review Board of the study institution (IRB number K15RISI0194).

### Endoscopic resection and follow-up

Gastric atrophy was defined with endoscopic evaluation and categorized as closed or open type using Kimura–Takemoto classification [20]. The lesion was demarcated after spraying indigo carmine solution, followed by saline injection and mucosal incision. A snare was used during EMR, while a Hook knife (Olympus Medical Systems Co. Ltd., Tokyo, Japan) was used during ESD. Electronic thermocoagulation was then applied simultaneously. Every procedure was were undergone by endoscopists who had performed these more than 5 years.

After ER, all patients underwent follow-up endoscopies within 6 months, at 12 months, and yearly thereafter. Suspicious mucosal lesions were biopsied and histologically evaluated in follow-up endoscopies. Follow-up endoscopies were done by the same endoscopists who had executed therapeutic procedures. Metachronous recurrence was defined as a new neoplasm that developed more than 1 year after ER.

### Determination of *H. pylori* status

*Helicobacter pylori* infection was confirmed with either a positive result on a rapid urease test (CLO test; Ballard Medical Products, Draper, Utah) or Warthin–Starry silver staining at the time of ER. After diagnosis, we used a 7-day regimen with a proton pump inhibitor, 1 g of amoxicillin and 500 mg of clarithromycin twice daily as a first-line eradication. If this regimen failed, a second-line prescription containing a proton pump inhibitor bid, 250 mg of metronidazole tid, 500 mg of tetracycline qid, and 240 mg of bismuth qid were administered for 14 days. A ^13^C-urea breath test (Helifinder; Medichems, Seoul, South Korea) was performed to find out whether eradication was successful at least 4 weeks after the treatment.

### Outcome measurements

Primary and secondary outcomes were the development of gastric neoplasm and gastric cancer after ER, respectively. Censored time was the day of last follow-up examination. Recurrence period was determined as the interval from the treatment to the last follow-up.

### Statistical analysis

Continuous data are presented as mean ± standard deviation or median (interquartile range), and categorical data as quantities and proportions. Continuous variables were compared using two-sample independent *t* test or Mann–Whitney U test for numerical variables, and nominal variables using Chi-square test or Fisher’s exact test. We performed the Kaplan–Meier method to analyze the cumulative incidence of gastric cancer and neoplasia after ER, and log-rank test for two-group comparison. We used Cox proportional hazard models for analyses of the independent association between pathologic results of gastric neoplasia and recurrence of metachronous tumor. Age, sex, presence of synchronous neoplasm, endoscopic atrophy, pathology of resected specimen, and *H. pylori* status were chosen as covariates for multivariate analysis. Statistical analysis was performed using SAS version 9.3 (SAS Institute, Cary, NC, USA) with a significance level of *P* < 0.05.

## Results

### Study population

**Fig. 1:**
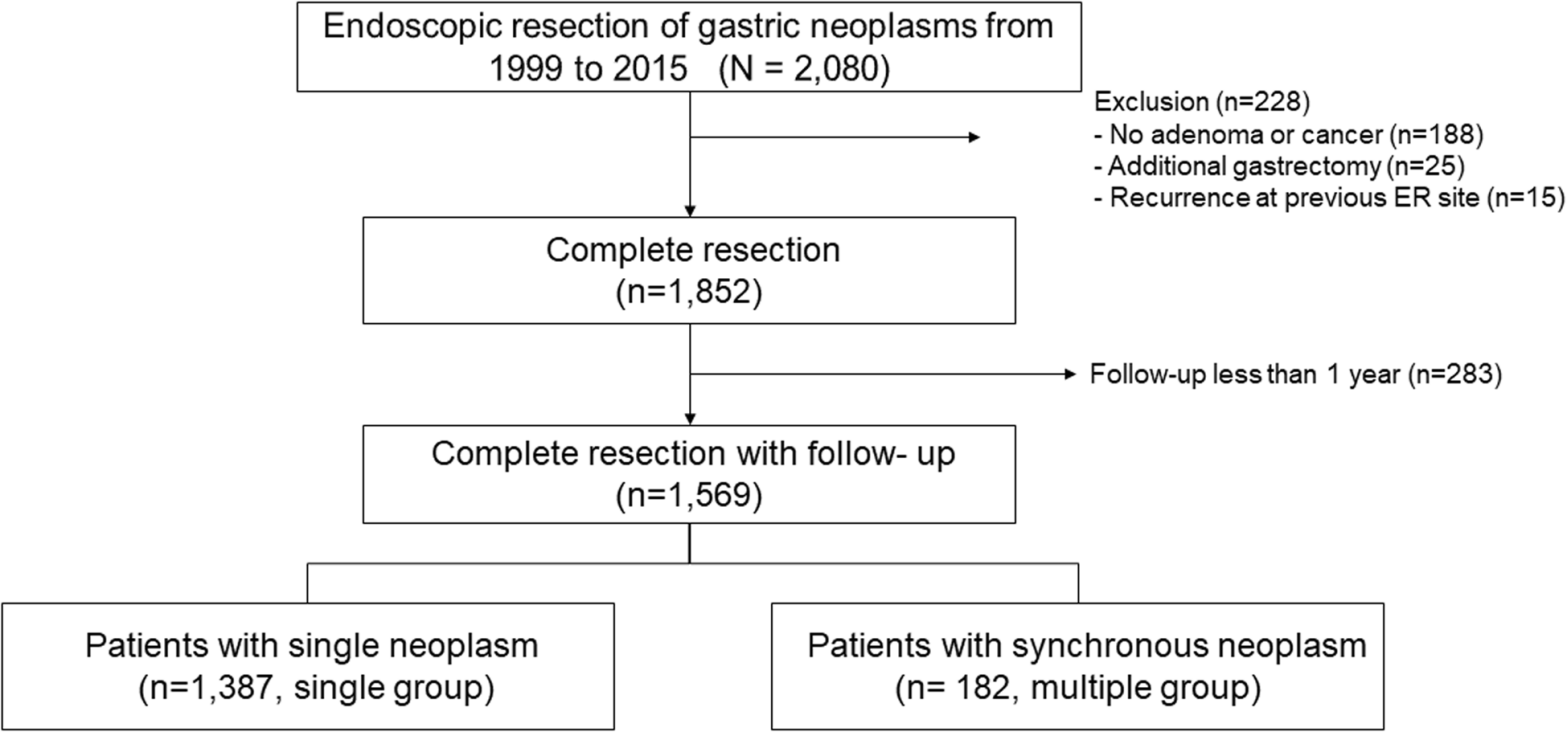
Flowchart of the study

A total of 2080 patients underwent ER for gastric neoplasms from January 1999 to December 2015. Of these patients, 511 patients were excluded due to additional gastrectomy for non-curative ER (*n* = 25), recurrence at previous ER site (*n* = 15), no diagnosis of adenoma or EGC (*n* = 188) and follow-up of less than 1 year (*n* = 283). After exclusion, 1569 patients were analyzed in the study (Fig. 1).

The baseline characteristics of the patients are summarized in Table 1. ER was performed in patients with EGCs (*n* = 678, 43.2%) and adenomas (*n* = 891, 56.8%). The median follow-up period was 30 months (range, 12–161 months). Among them, 1387 (88.4%) were in the single group and 182 patients (11.6%) were in the multiple group. The median age was higher in the multiple than the single group (66.1 ± 8.0 vs. 63.2 ± 9.4 years, *P* < 0.001). However, there were no significant differences in other baseline characteristics including sex, proportion of initial pathology, location, invasion depth of tumors, incidence of *H. pylori* infection, and follow-up duration between the two groups (Table 1). In the single group, cancer pathology included differentiated cancer (*n* = 576, 41.5%) and undifferentiated cancer (*n* = 22, 1.6%). In the multiple group, cancer pathology included differentiated cancer (*n* = 80, 44.0%) and no undifferentiated cancer (*n* = 0. 0%).

**Table 1:** Baseline characteristics in single and multiple groups

	Total (*N* = 1569)	Single group (*n* = 1387)	Multiple group (*n* = 182)	*P*
Age (mean ± SD)	63.2 ± 9.3	63.2 ± 9.4	66.1 ± 8.0	< 0.001
Male, n (%)	1103 (70.3%)	965 (69.6%)	138 (75.8%)	0.078
Cancer, n (%)	678 (43.2%)	598 (43.1%)	80 (44.0%)	0.829
Endoscopic atrophy				0.016
Open type	895 (57.0%)	776 (56.0%)	119 (65.4%)	
Closed type	674 (43.0%)	611 (44.1%)	63 (34.6%)	
Tumor location				0.545
Upper, n (%)	73 (4.7%)	66 (4.7%)	7 (3.9%)	
Middle, n (%)	491 (31.3%)	428 (30.9%)	63 (34.6%)	
Lower, n (%)	1005 (64.1%)	893 (64.4%)	112 (61.5%)	
Macroscopic type				0.384
Depressed, n (%)	222 (14.1%)	204 (14.6%)	18 (10.7%)	
Lesion diameter, mm (mean ± SD)	14.5 ± 10.8	14.5 ± 11	15 ± 9.5	0.496
Depth of invasion				0.291
Mucosa, n (%)	1522 (9.1%)	1343 (96.8%)	179 (98.4%)	
Submucosa, n (%)	47 (3.0%)	44 (3.2%)	3 (1.7%)	
*Helicobacter pylori* infection, n (%)^a^	793/1501 (52.8%)	701/1323 (53.0%)	92/178 (51.7%)	0.714
Persistent	261 (36.3%)	227 (17.2%)	34 (19.1%)	
Eradicated	532 (68.4%)	474 (35.8%)	58 (32.6%)	
Negative	708 (95.3%)	622 (47.0%)	86 (48.3%)	
Follow-up, months (median, range)	30 (12–161)	30 (12–161)	25 (12–112)	0.261

^a^68 patients were not examined

### Incidence of metachronous neoplasm after ER

**Fig. 2:**
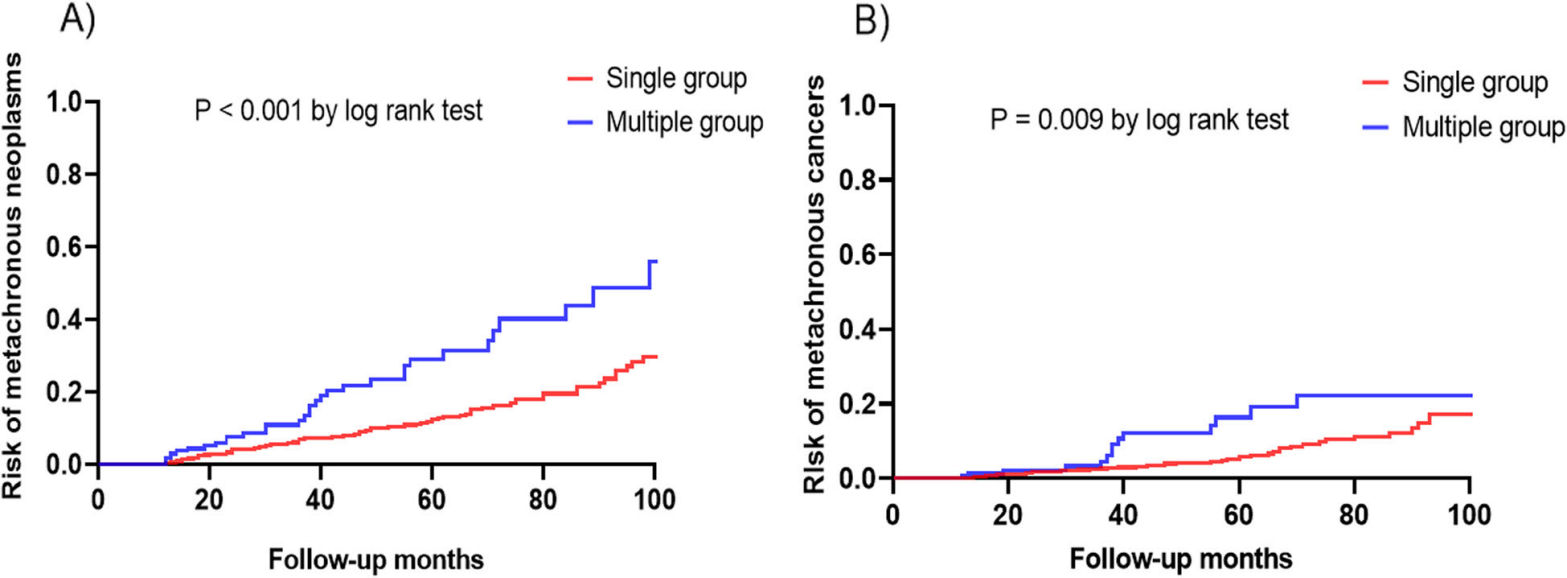
Kaplan-Meier analyses of cumulative incidence of metachronous neoplasm and cancer after endoscopic resection of gastric neoplasm (adenoma and EGC). **a**, Cumulative incidence of gastric neoplasm. **b**, Cumulative incidence of gastric cancer. EGC, early gastric cancer; ER, endoscopic resection

During the follow-up period, metachronous recurrence was found in 152 patients (9.7%). All metachronous neoplasms were successfully treated by ER without additional treatment. In the single group, 119 patients (8.6%) had metachronous neoplasms with pathologies revealed as 65 adenomas (4.6%) and 54 cancers (3.9%). In the multiple group, metachronous neoplasms were found in 33 patients (18.1%) with pathologies revealed as 19 adenomas (10.4%) and 14 cancers (7.7%). Kaplan-Meier analysis of the cumulative incidence of metachronous neoplasm and cancers after ER showed a significant difference between the two groups (*P* < 0.01, log-rank test). (Fig. 2a and b). Table 2 shows the results of univariate and multivariate Cox proportional hazards models for the risk of metachronous gastric neoplasm. On univariate analysis, multiple group (hazard ratio [HR], 2.32 95% confidence interval [CI], 1.54–3.40) and persistent *H. pylori* infection compared to eradicated *H. pylori* infection (hazard ratio [HR], 1.64; 95% confidence interval [CI], 1.04–2.58) were significant factors associated with the development of metachronous neoplasm. In addition, the multiple group was also a significant factor associated with the development of metachronous cancer (HR, 2.2; 95% CI, 1.19–3.88) However, the pathology of the resected specimen, severity of atrophic gastritis, and patient demographics were not significant factors for metachronous neoplasia. Multivariate Cox analysis shows that the multiple group (hazard ratio [HR], 2.40; 95% confidence interval [CI], 1.62–3.34) and persistent *H. pylori* infection (hazard ratio [HR], 1. 71; 95% confidence interval [CI], 1.09–2.61) were significant factors associated with the development of metachronous neoplasia.

**Table 2:** Cox proportional hazard models for the cumulative incidence of metachronous neoplasm after endoscopic resection in patients with and without synchronous neoplasms

	Univariate analysis			Multivariate analysis		
	HR	95% CI	*P*	HR	95% CI	*P*
Age, years	1.02	0.99–1.04	0.984			
Male vs. female	1.12	0.82–1.72	0.380			
Multiple vs. Single group	2.32	1.54–3.40	< 0.001	2.40	1.62–3.34	< 0.001^a^
Open vs. Closed type of atrophic gastritis	1.20	0.86–1.70	0.288			
EGC vs. Adenoma	1.01	0.73–1.40	0.952			
*Helicobacter pylori*^a^						
Eradicated	1					
Negative	1.15	0.79–1.68	0.466			
Persistent	1.64	1.04–2.58	0.032	1.71	1.09–2.61	0.002^a^

^a^68 patients were not examined

Abbreviations: *HR* hazard ratio, *CI* confidence interval, *EGC* early gastric cancer

### Characteristics of metachronous neoplasm in the single and the multiple group

The clinicopathological characteristics of metachronous gastric neoplasm are summarized in Table 3. There was a significant difference in patient age and no significant difference in sex, pathological characteristics, infection and eradication rate of *H. pylori*, and interval time to detect metachronous neoplasm (Table 3).

**Table 3:** Clinicopathologic characteristics of metachronous neoplasm after endoscopic resection of gastric neoplasm between the single and the multiple group

	Single group (*n* = 119)	Multiple group (*n* = 33)	*P*
Age (median, range)	63 (44–83)	66 (48–81)	0.035
Gender			0.965
Male, n (%)	87 (73.1%)	24 (72.7%)	
Female, n (%)	32 (26.9%)	9 (27.3%)	
Cancer, n (%)	62 (52.1%)	17 (51.5%)	0.952
Tumor location			0.414
Upper, n (%)	6 (5.0%)	0 (0%)	
Middle, n (%)	36 (30.3%)	10 (30.3%)	
Lower, n (%)	77 (64.7%)	23 (69.7%)	
Macroscopic type			0.197
Depressed, n (%)	13 (10.9%)	0 (0%)	
Diameter, cm (median, range)	12 (2–65)	14 (1–36)	0.411
Depth of invasion			0.954
Mucosa, n (%)	114 (95.8%)	32 (97%)	
Submucosa, n (%)	5 (4.2%)	1 (3.0%)	
*Helicobacter pylori*			
Positive rate, n (%)	61 (51.3%)	14 (42.4%)	0.579
Eradication rate, n (%)	36 (30.5%)	8 (24.2%)	0.512
Time to recur (months), (median and range)	47 (12–155)	24 (12–100)	0.451

### The incidence and characteristics of metachronous neoplasm in the multiple group

**Fig. 3:**
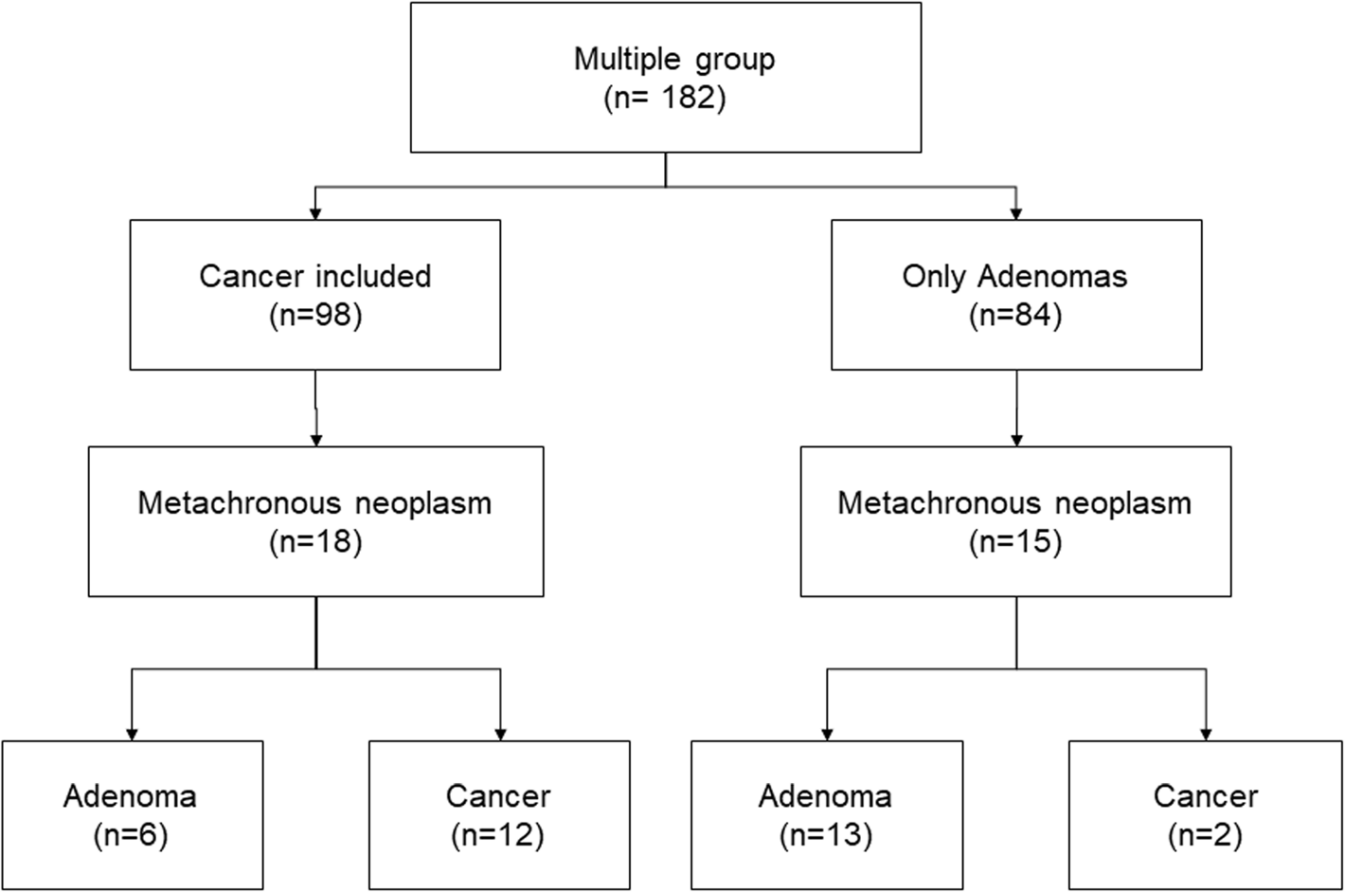
Metachronous recurrence in the multiple group (*n* = 182)

**Fig. 4:**
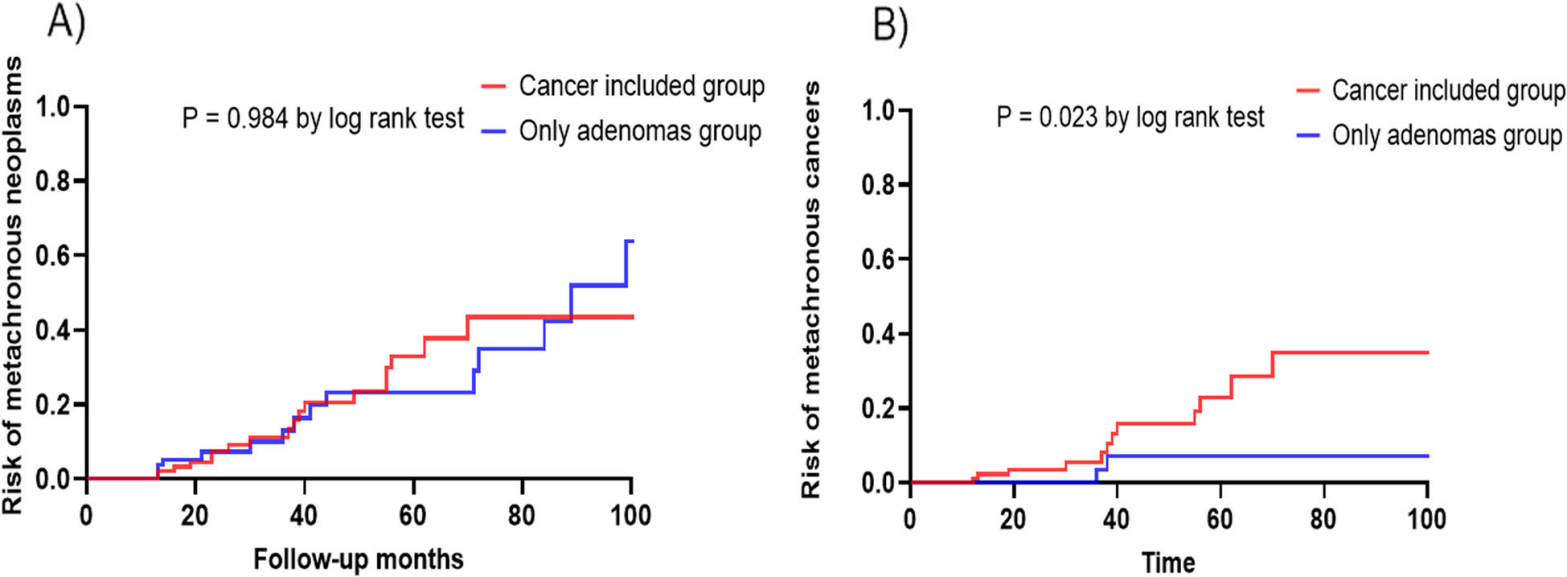
Kaplan-Meier analysis of cumulative incidence metachronous neoplasm and cancer between cancer-included group and only adenomas groups. **a**, Cumulative incidence of gastric neoplasm. **b**, Cumulative incidence of gastric cancer. EGC, early gastric cancer; ER, endoscopic resection

To compare the incidence of metachronous neoplasm depending on pathology, the multiple group was further divided into patients with and without cancer in multiple lesions. There were no significant differences in patients’ characteristics, endoscopic findings, and *H. pylori* infection between the cancer-included and the adenoma-only groups. Metachronous neoplasm was found in 18 (18%) patients in the cancer-included group and 15 (18%) patients in the only-adenomas group (Fig. 3), which was not statistically significant (*P* = 0.984, log-rank test; Fig. 4a). However, the proportion of recurred pathology was different between the cancer-included and the only-adenomas subgroups in the multiple group. Among metachronous neoplasms (*n* = 18) of the cancer-included subgroup, six adenomas (33%, five low grade and one high grade) and 12 differentiated cancers (67.0%) were found during the follow-up period. Among metachronous neoplasms (*n* = 15) of the only-adenomas subgroup, 13 adenomas (87%, ten low grade and three high grade) and two differentiated cancers (13.0%) were found. The cumulative incidence of metachronous cancers after ER showed a significant difference between the two subgroups (*P* = 0.023, log-rank test; (Fig. 4b).

## Discussion

In our study, we compared the risk of metachronous recurrence between patients with and without synchronous gastric neoplasm after ER. Among all patients with gastric neoplasms, 11.6% had synchronous lesions. Patients with synchronous gastric neoplasms had about two-fold higher recurrence risk of neoplasms compared to those without synchronous lesions. Among patients with synchronous neoplasms, recurrence rate was not significantly different between patients with and without cancer. However, cancer recurrence rate was higher in patients with cancers than those with only adenomas.

Metachronous gastric cancer from remnant stomach after ER has been an important problem, although ER can preserve the stomach and contribute to a better quality of life compared to surgery [10, 11]. There have been many studies evaluating the risk factors of metachronous gastric cancer after ER. Previous studies usually focused on metachronous recurrence after ER of EGC and incidence of metachronous cancer not including adenomas. In this study, we investigated the recurrence of adenoma as well as cancer due to the higher possibility of development of invasive carcinomas from adenomas after ER [8, 9, 21]. We also investigated tumor recurrence not only in EGC patients but also in adenoma patients based on our previous report [16].

It is important to evaluate the risk factors of metachronous neoplasm to predict prognosis and determine the follow-up strategy. In previous studies, older age, persistent *H. pylori* infection, and severity of atrophy were associated with a higher risk of metachronous gastric cancer [13–15, 22, 23]. In this study, the significantly higher incidence of metachronous neoplasm was found in patients with synchronous neoplasms, suggesting the importance of meticulous examinations in these patients. Persistent *H. pylori* infection was also associated with increase of metachronous neoplasm which were concordant with previous studies [13–15]. However, the severity of atrophy was not associated with metachronous neoplasm incidence in the present study. This might be caused by the difference in *H. pylori* infection rate and different outcome (adenoma as well as cancer) compared to previous studies [22, 23]. We evaluated the degree of atrophy only with white light image, which is more associated with higher intra-observer and inter-observer variability than image-enhanced endoscopies like autofluorescence image [24].

Our study further strengthened the importance of pathology in multiple neoplasms because the incidence of metachronous cancer was higher in patients with multiple neoplasms including cancer, as shown in Fig. 4b. Therefore, we speculate that patients with multiple gastric neoplasm should have regular meticulous endoscopy follow-up during the follow-up period. Furthermore, we also consider the higher possibility of metachronous gastric cancer in the cancer-included group more than the only adenoma group in multiple gastric neoplasm.

Strict follow-up strategy has several aspects including follow-up interval, examination time of endoscopy, endoscopy methods, and additional considerations. Even though the follow-up interval seems to be the most important among these, there has been no report concerning individualized interval according to risk factors. There is no established guidance on the interval and duration of endoscopic surveillance for recurrence of metachronous gastric cancer [12, 25, 26]. The Japanese guideline recommended annual or biannual endoscopy without specifying the duration [27]. There have been studies suggesting intensive surveillance performed biannually in the first year to detect missed concomitant invasive cancers [25, 28]. In our study, all patients after ER of gastric neoplasm including adenoma were scheduled to be examined with endoscopy biannually at the first year and annually thereafter. The same scheduled examination was performed in patients who were diagnosed to have EGC or adenoma. Considering our results, patients with multiple synchronous gastric neoplasms should have meticulous examination during the follow-up period to find metachronous lesions.

In the aspect of meticulous examination, a few studies have reported the importance of observation time in screening or symptom-based settings [29, 30]. We reported the importance of longer observation time to find possible synchronous lesions [31]. We also investigated the new endoscopic imaging technique, which was related to a higher detection rate of synchronous neoplasms than conventional white-light image endoscopy [32]. Based on the present study, the detection of synchronous neoplasms is important in predicting the risk of metachronous gastric neoplasms. Further studies are needed to address these possibilities.

The exact mechanism of the significantly higher incidence of metachronous recurrence in patients with multiple gastric neoplasms than those with a single lesion remains unclear. This result may be explained by a cancer field effect. The concept of the field effect in cancer is described as a morphologically normal epithelium developing into a tumor as a result of expansion of a genetically abnormal clone or epigenetic alterations, which include hypermethylation of the DNA promoter of certain tumor suppressor genes [33]. This theory has been explored through the association between cancer development and methylation status in the gastric mucosa [34–36]. Patients with multiple gastric cancers had significantly higher methylation levels of cancer-related genes than those with a single gastric cancer [37–39]. The mechanisms of field effect in gastric cancer were explained by the expansion and spread of mutated gastric stem cells. A previous study showed that human gastric body units are clonal, contain multiple multipotential stem cells, and provided definitive evidence for how mutations spread within the human stomach, and how field cancerization develops [40]. However, we do not know the exact mechanism of the development of multiple gastric neoplasms.

There are several limitations to our study. First, we should acknowledge that there was a wide variation of follow-up period (ranging from 12 to 131 months), and that the median follow-up period of 30 months was not long enough for evaluation of metachronous lesions. A further prospective long-term study that assesses the development of metachronous cancer after ER is required to confirm the findings of our study. Second, we did not take multiple samples to evaluate the extent of atrophy. Third, the number of metachronous neoplasm in the subgroup was too small to show a significant difference in recurrence between the cancer-included group and the only adenomas group. Further studies on a larger scale are needed to evaluate the significant difference in recurrence according to the pathology of the multiple group. Finally, this study did not specifically investigate the appropriate follow-up period after ER.

## Conclusion

This study showed that patients with synchronous gastric neoplasms, including adenomas, had a higher risk of metachronous neoplasms than patients with a single neoplasm. The incidence of metachronous cancer was higher in subgroups with cancer than subgroups with adenomas only. Therefore, we should consider a strict follow-up strategy with meticulous endoscopic examination in the presence of synchronous neoplasms, especially if cancer is detected in at least one lesion.

## Data Availability

The datasets generated or analyzed during the current study are available from the corresponding author on reasonable request.

## Abbreviations

CI: Confidence interval
EGC: Early gastric cancer
EMR: Endoscopic mucosal resection
ESD: Endoscopic submucosal dissection
ER: Endoscopic resection
*H. pylori*: *Helicobacter pylori*
HR: Hazard ratio
